# Supplementary figures and images for: A bacterial type III effector hijacks plant ubiquitin proteases to evade degradation
Source: PLoS Pathog. 2025 Jan 22;21(1):e1012882. doi: 10.1371/journal.ppat.1012882 (PMC11771917; doi:10.1371/journal.ppat.1012882)

**A**

## B

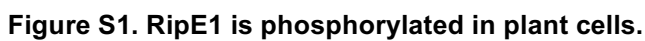

(B) Representative mass spectra of the phosphorylated peptides shown in (A).

Supplement: S1 Fig — (A) Phosphorylated peptides detected after immunoprecipitation of RipE1-GFP in N. benthamiana leaves followed by LC-MS/MS analysis. The number of the residues, peptide sequences, and mascot ion scores are shown. Phosphorylated residues are shown in red. (B) Representative mass spectra of the phosphorylated peptides shown in (A). (PDF) [file ppat.1012882.s001.pdf]
