# Supplementary material for: A bacterial type III effector hijacks plant ubiquitin proteases to evade degradation
Source: PLoS Pathog. 2025 Jan 22;21(1):e1012882. doi: 10.1371/journal.ppat.1012882 (PMC11771917; doi:10.1371/journal.ppat.1012882)
Supplement: S2 Fig — (A) Immunoprecipitation assay to determine the ubiquitination status of RipE1 and phosphodeficient mutants. Agrobacterium carrying the indicated constructs were infiltrated as in Fig 1H. Samples were collected 30 hpi, before the appearance of cell death. Given the different RipE1 variants show different protein accumulation, different volumes of the protein samples were loaded to show a comparable RipE1-GFP protein accumulation between different lanes after immunoprecipitation, allowing the detection of ubiquitination in the same amount of RipE1 protein. The relative loading volumes and protein abundance are indicated above lanes. Anti-GFP beads were used for immunoprecipitation. An anti-ubiquitin (P4D1) antibody was used to detect ubiquitinated proteins. Protein marker sizes are shown for reference. This experiment was repeated 3 times, and the quantification of the different repeats is shown in (B). (B) Quantification of the relative protein ubiquitination of the different repeats of the assay shown in (A), measured using Image J. Ubiquitination values were normalized using the respective protein accumulation and represented as relative to the GFP control for each repeat. Values indicate mean ± SE (n = 3 biological replicates). Different letters indicate significant differences (one-way ANOVA, Tukey’s test, p < 0.05). P values are shown for reference. (C) Composite data representation of all the replicates shown in Figs 1H and S2A. Values indicate mean ± SE (n = 6 biological replicates). Different letters indicate significant differences (one-way ANOVA, Tukey’s test, p < 0.05). (PDF) [file ppat.1012882.s002.pdf]

Figure S2

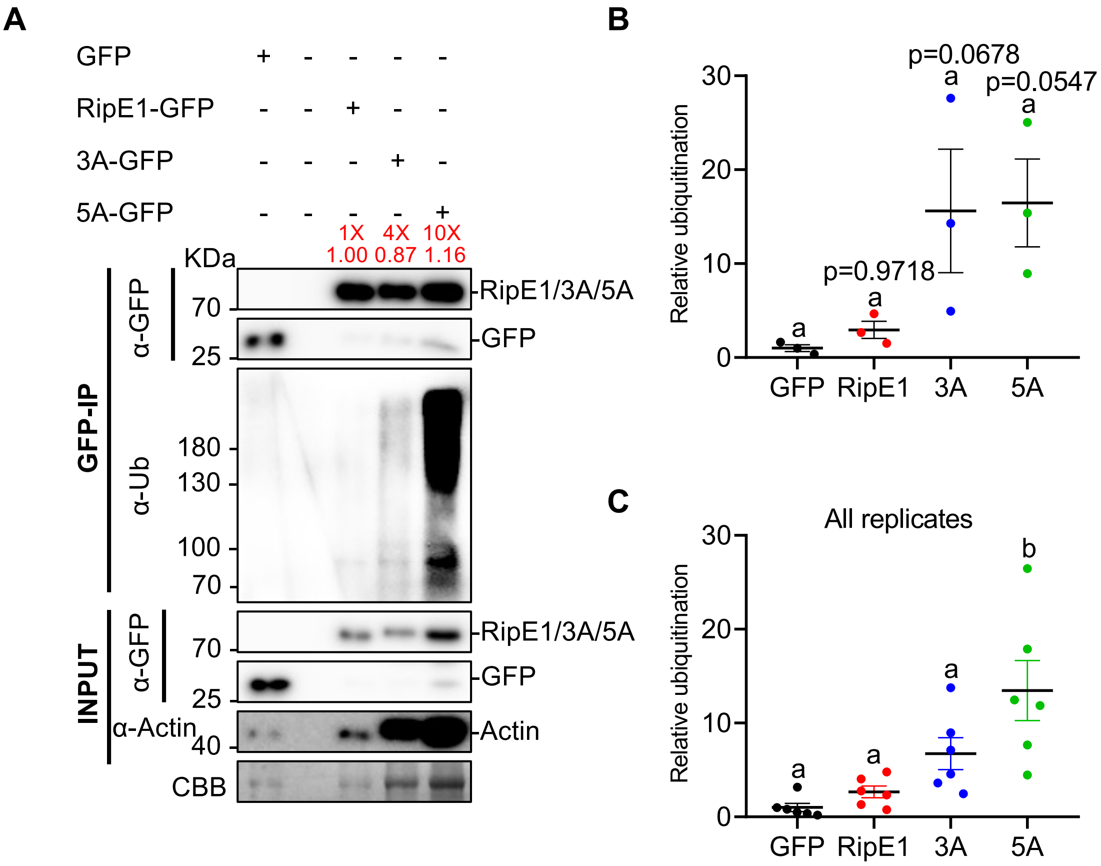

Figure S2. RipE1 phosphorylation counteracts its ubiquitination.

(A) Immunoprecipitation assay to determine the ubiquitination status of RipE1 and phosphodeficient mutants. Agrobacterium carrying the indicated constructs were infiltrated as in Figure 1E. Samples were collected 30 hpi, before the appearance of cell death. Given the different RipE1 variants show different protein accumulation, different volumes of the protein samples were loaded to show a comparable RipE1-GFP protein accumulation between different lanes after immunoprecipitation, allowing the detection of ubiquitination in the same amount of RipE1 protein. The relative loading volumes and protein abundance are indicated above lanes. Anti-GFP beads were used for immunoprecipitation. An anti-ubiquitin (P4D1) antibody was used to detect ubiquitinated proteins. Protein marker sizes are shown for reference. This experiment was repeated 3 times, and the quantification of the different repeats is shown in (B).

(B) Quantification of the relative protein ubiquitination of the different repeats of the assay shown in (A), measured using Image J. Ubiquitination values were normalized using the respective protein accumulation and represented as relative to the GFP control for each repeat. Values indicate mean ± SE (n = 3 biological replicates). Different letters indicate significant differences (one-way ANOVA, Tukey's test, $p < 0.05$). P values are shown for reference.

(C) Composite data representation of all the replicates shown in Figure 1E and S2A. Values indicate mean ± SE (n = 6 biological replicates). Different letters indicate significant differences (one-way ANOVA, Tukey's test, $p < 0.05$).
