# Supplementary material for: A bacterial type III effector hijacks plant ubiquitin proteases to evade degradation
Source: PLoS Pathog. 2025 Jan 22;21(1):e1012882. doi: 10.1371/journal.ppat.1012882 (PMC11771917; doi:10.1371/journal.ppat.1012882)
Supplement: S3 Fig — (A) Confocal microscopy images showing the subcellular localization of free GFP and RipE1-GFP. RipE1-GFP or GFP (as control) were expressed in 4-week-old N. benthamiana using Agrobacterium (OD600 = 0.5). Microscopy images were captured 48 hours post-inoculation. A size bar (25 μM) is shown for reference. The right panel shows a western blot to verify the accumulation of these proteins, ruling out significant GFP cleavage in the RipE1-GFP samples. Blots were analyzed using an anti-GFP antibody, and protein marker sizes are shown for reference. (B) Phylogenetic tree showing the NbUCH proteins identified in this work, together with all the other proteins annotated as UCH in the N. benthamiana proteome, showing at least 50% identity when compared with NbUCH05, NbUCH12, and NbUCH15. The tree includes also the proteins encoded by the closest Arabidopsis orthologs, namely AtUBP12 and AtUBP13. (C) Confocal microscopy images showing the subcellular localization of RipE1 and NbUCH15 in N. benthamiana. RipE1-RFP or RFP (as control) were co expressed with NbUCH15-GFP or GFP (as control) using Agrobacterium (final OD600 = 0.5). Microscopy images were captured 46 hours post-inoculation. Sequential scanning was used to avoid interference between the GFP and RFP channels. A size bar (10 μM) is shown for reference. Each experiment was repeated 3 with similar results. The right panels show the quantification of the fluorescent signals in the boxed areas. (D) Western blot to verify the accumulation of the proteins in (C). Blots were analyzed using an anti-GFP and anti-RFP antibodies, and protein marker sizes are shown for reference. (E) Western blot to verify the accumulation of the proteins in the FRET-FLIM experiments shown in Fig 2C. Blots were analyzed using an anti-GFP and anti-RFP antibodies, and protein marker sizes are shown for reference. An anti-actin antibody was used to verify equal loading. (PDF) [file ppat.1012882.s003.pdf]

Figure S3

A

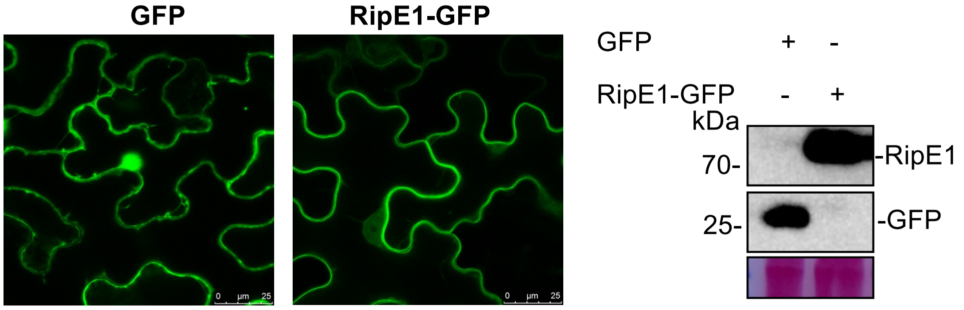
GFP
RipE1-GFP
GFP + -
RipE1-GFP - +
kDa
70-
25-
-RipE1
-GFP

B

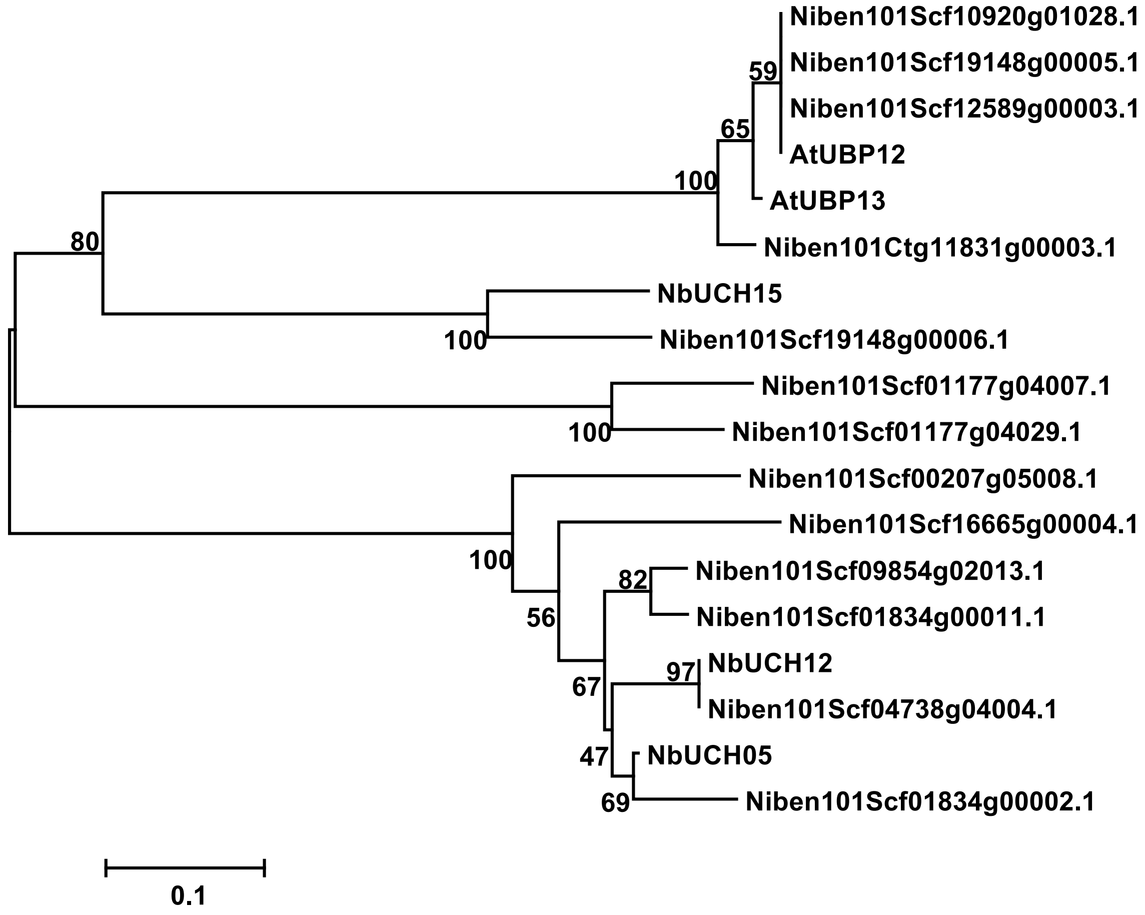
Niben101Scf10920g01028.1
Niben101Scf19148g00005.1
Niben101Scf12589g00003.1
AtUBP12
AtUBP13
Niben101Ctg11831g00003.1
NbUCH15
Niben101Scf19148g00006.1
Niben101Scf01177g04007.1
Niben101Scf01177g04029.1
Niben101Scf00207g05008.1
Niben101Scf16665g00004.1
Niben101Scf09854g02013.1
Niben101Scf01834g00011.1
NbUCH12
Niben101Scf04738g04004.1
NbUCH05
Niben101Scf01834g00002.1
59
65
100
80
100
100
100
82
56
97
67
47
69
0.1

Figure S3
(continue)

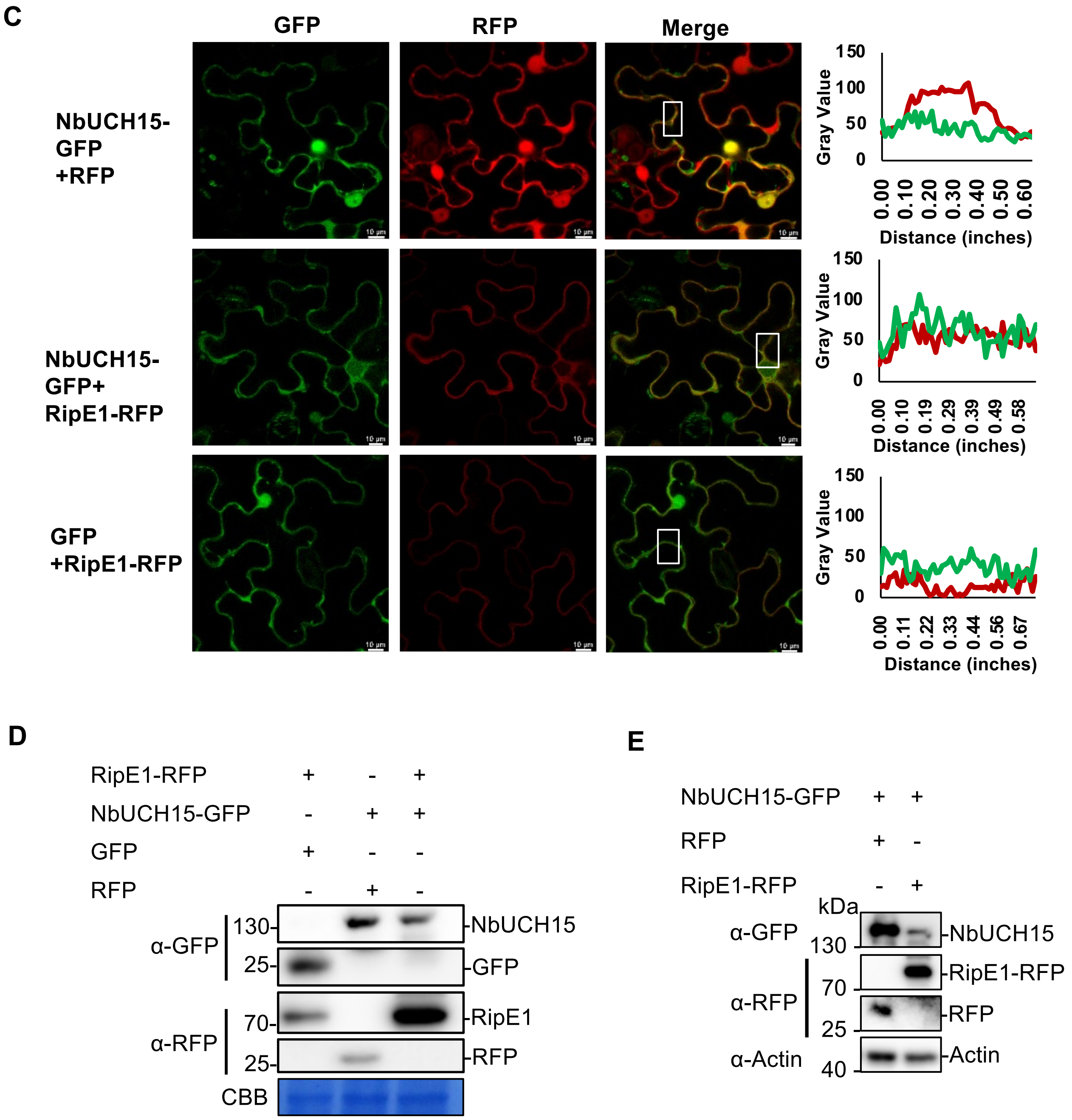

Figure S3. RipE1 and NbUCH15 interact and partially co-localize in the cell periphery and nucleus.

(A) Confocal microscopy images showing the subcellular localization of free GFP and RipE1-GFP. RipE1-GFP or GFP (as control) were expressed in 4-week-old *N. benthamiana* using Agrobacterium (OD_{600}=0.5). Microscopy images were captured 48 hours post-inoculation. A size bar (25 μM) is shown for reference. The right panel shows a western blot to verify the accumulation of these proteins, ruling out significant GFP cleavage in the RipE1-GFP samples. Blots were analyzed using an anti-GFP antibody, and protein marker sizes are shown for reference.

(B) Phylogenetic tree showing the NbUCH proteins identified in this work, together with all the other proteins annotated as UCH in the *N. benthamiana* proteome, showing at least 50% identity when compared with NbUCH05, NbUCH12, and NbUCH15. The tree includes also the proteins encoded by the closest Arabidopsis orthologs, namely AtUBP12 and AtUBP13.

(C) Confocal microscopy images showing the subcellular localization of RipE1 and NbUCH15 in *N. benthamiana*. RipE1-RFP or RFP (as control) were co expressed with NbUCH15-GFP or GFP (as control) using Agrobacterium (final OD_{600}=0.5). Microscopy images were captured 46 hours post-inoculation. A size bar (10 μM) is shown for reference. Each experiment was repeated 3 with similar results. The right panels show the quantification of the fluorescent signals in the boxed areas.

(D) Western blot to verify the accumulation of the proteins in (C). Blots were analyzed using an anti-GFP and anti-RFP antibodies, and protein marker sizes are shown for reference.

(E) Western blot to verify the accumulation of the proteins in the FRET-FLIM experiments shown in Figure 2C. Blots were analyzed using an anti-GFP and anti-RFP antibodies, and protein marker sizes are shown for reference. An anti-actin antibody was used to verify equal loading.
