# Supplementary material for: A bacterial type III effector hijacks plant ubiquitin proteases to evade degradation
Source: PLoS Pathog. 2025 Jan 22;21(1):e1012882. doi: 10.1371/journal.ppat.1012882 (PMC11771917; doi:10.1371/journal.ppat.1012882)
Supplement: S4 Fig — (A) Quantitative RT-PCR to determine the expression of NbUCH05, NbUCH12, and NbUCH15 in the experiments shown in Fig 3. Samples were taken 4 or 8 dpi. Expression values are relative to the expression of the housekeeping gene NbEF1a. Values indicate mean ± SE (n = 3 biological replicates). (B) Quantitative RT-PCR to determine the expression (silencing efficiency) of NbPtr1. Samples were taken 8 dpi. Expression values are relative to the expression of the housekeeping gene NbEF1a. Values indicate mean ± SE (n = 3 biological replicates). Each experiment was repeat at least three times with similar results. (PDF) [file ppat.1012882.s004.pdf]

Figure S4

A

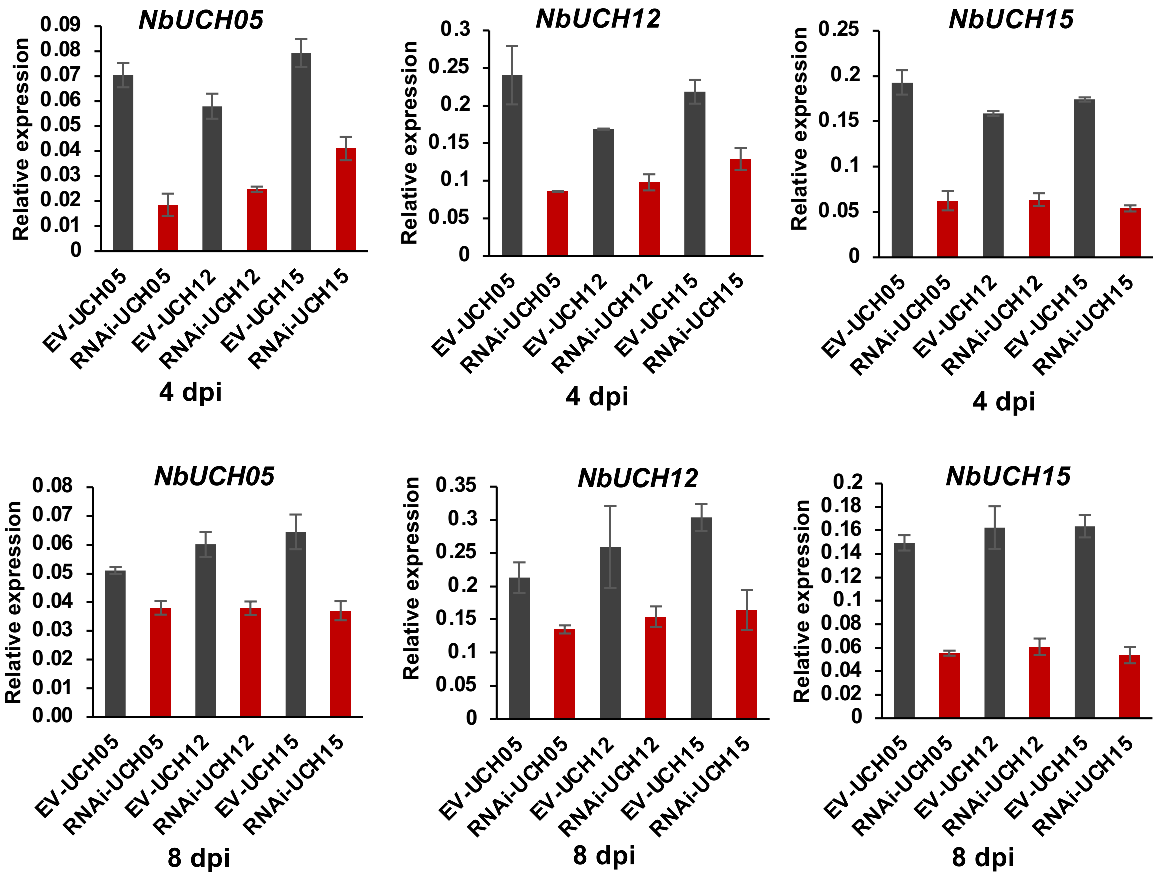

B

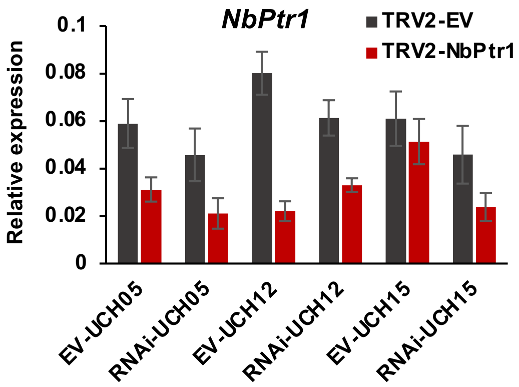

Figure S4. Validation of the silencing efficiency in RNAi and VIGS assays.

(A) Quantitative RT-PCR to determine the expression of *NbUCH05*, *NbUCH12*, and *NbUCH15* in the experiments shown in Figure 3. Samples were taken 4 or 8 dpi. Expression values are relative to the expression of the housekeeping gene *NbEF1a*. Values indicate mean ±SE (n=3 biological replicates).

(B) Quantitative RT-PCR to determine the expression (silencing efficiency) of *NbPtr1*. Samples were taken 8 dpi. Expression values are relative to the expression of the housekeeping gene *NbEF1a*. Values indicate mean ±SE (n=3 biological replicates).

Each experiment was repeat at least three times with similar results.
