# Supplementary material for: A bacterial type III effector hijacks plant ubiquitin proteases to evade degradation
Source: PLoS Pathog. 2025 Jan 22;21(1):e1012882. doi: 10.1371/journal.ppat.1012882 (PMC11771917; doi:10.1371/journal.ppat.1012882)
Supplement: S5 Fig — (A) Quantitative RT-PCR to determine the expression of RipE1 and RipAA in N. benthamiana tissues in the experiment shown in Fig 5A. Expression values are relative to the expression of the housekeeping gene NbEF1a. Values indicate mean ± SE (n =9 biological replicates). Composite data from 3 independent biological replicates. (PDF) [file ppat.1012882.s005.pdf]

Figure S5

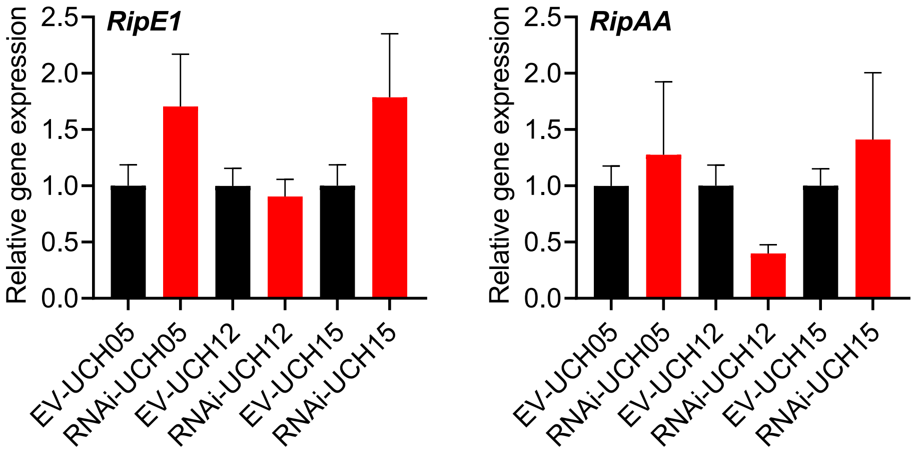

Figure S5. The expression of *RipE1* or *RipAA* is not significantly altered by silencing of *NbUCH* genes.

(A) Quantitative RT-PCR to determine the expression of *RipE1* and *RipAA* in *N. benthamiana* tissues in the experiment shown in Figure 5A. Expression values are relative to the expression of the housekeeping gene *NbEF1a*. Values indicate mean ± SE (n =9 biological replicates). Composite data from 3 independent biological replicates.
