# Supplementary material for: A bacterial type III effector hijacks plant ubiquitin proteases to evade degradation
Source: PLoS Pathog. 2025 Jan 22;21(1):e1012882. doi: 10.1371/journal.ppat.1012882 (PMC11771917; doi:10.1371/journal.ppat.1012882)
Supplement: S6 Fig — (A) Immunoprecipitation assay to determine the ubiquitination status of RipE1 upon silencing of NbUCH15. Agrobacterium carrying the indicated constructs were infiltrated as in Fig 6A. Samples were collected 30 hpi, before the appearance of cell death. In this case, the same sample volumes were loaded into each lane, showing a reduced accumulation of RipE1, but nevertheless a stronger ubiquitination. Anti-GFP beads were used for immunoprecipitation. An anti-ubiquitin (P4D1) antibody was used to detect ubiquitinated proteins. The accumulation of native NbUCH proteins was detected using a custom anti-NbUCH antibody. This experiment was repeated 3 times, and the quantification of the different repeats is shown in (B). (B) Quantification of the relative protein ubiquitination of the different repeats of the assay shown in (A), measured using Image J. Ubiquitination values were normalized using the respective protein accumulation and represented as relative to the empty vector control for each repeat. Values indicate mean ± SE (n = 3 biological replicates). P values are shown for reference according to a Student´s t test. (C) Composite data representation of all the replicates shown in Figs 6A and S6A. Values indicate mean ± SE (n = 6 biological replicates). Asterisk indicates significant differences compared to the control according to a Student’s t test (* p < 0.05). (PDF) [file ppat.1012882.s006.pdf]

Figure S6

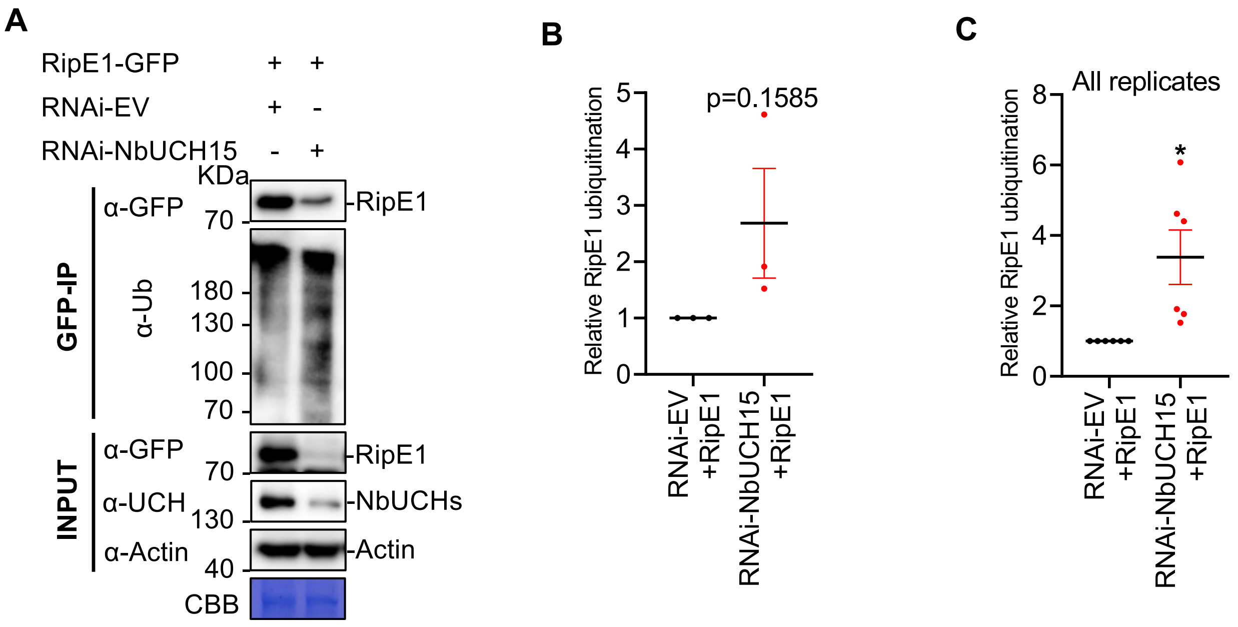

Figure S6. RipE1 ubiquitination is enhanced upon silencing of *NbUCH15*.

(A) Immunoprecipitation assay to determine the ubiquitination status of RipE1 upon silencing of *NbUCH15*. Agrobacterium carrying the indicated constructs were infiltrated as in Figure 6A. Samples were collected 30 hpi, before the appearance of cell death. In this case, the same sample volumes were loaded into each lane, showing a reduced accumulation of RipE1, but nevertheless a stronger ubiquitination. Anti-GFP beads were used for immunoprecipitation. An anti-ubiquitin (P4D1) antibody was used to detect ubiquitinated proteins. The accumulation of native NbUCH proteins was detected using a custom anti-NbUCH antibody. This experiment was repeated 3 times, and the quantification of the different repeats is shown in (B).

(B) Quantification of the relative protein ubiquitination of the different repeats of the assay shown in (A), measured using Image J. Ubiquitination values were normalized using the respective protein accumulation and represented as relative to the empty vector control for each repeat. Values indicate mean ± SE (n = 3 biological replicates). P values are shown for reference according to a Student´s t test.

(C) Composite data representation of all the replicates shown in Figure 6A and S6A. Values indicate mean ± SE (n = 6 biological replicates). Asterisk indicates significant differences compared to the control according to a Student's t test (* $p < 0.05$).
