# Supplementary material for: A bacterial type III effector hijacks plant ubiquitin proteases to evade degradation
Source: PLoS Pathog. 2025 Jan 22;21(1):e1012882. doi: 10.1371/journal.ppat.1012882 (PMC11771917; doi:10.1371/journal.ppat.1012882)
Supplement: S7 Fig — Protein sequence alignment showing RipE1 versions in different R. solanacearum strains. The phosphorylated residues analyzed in this study are indicated in red, together with the conserved domain A, and the catalytic sites. (PDF) [file ppat.1012882.s007.pdf]

Figure S7

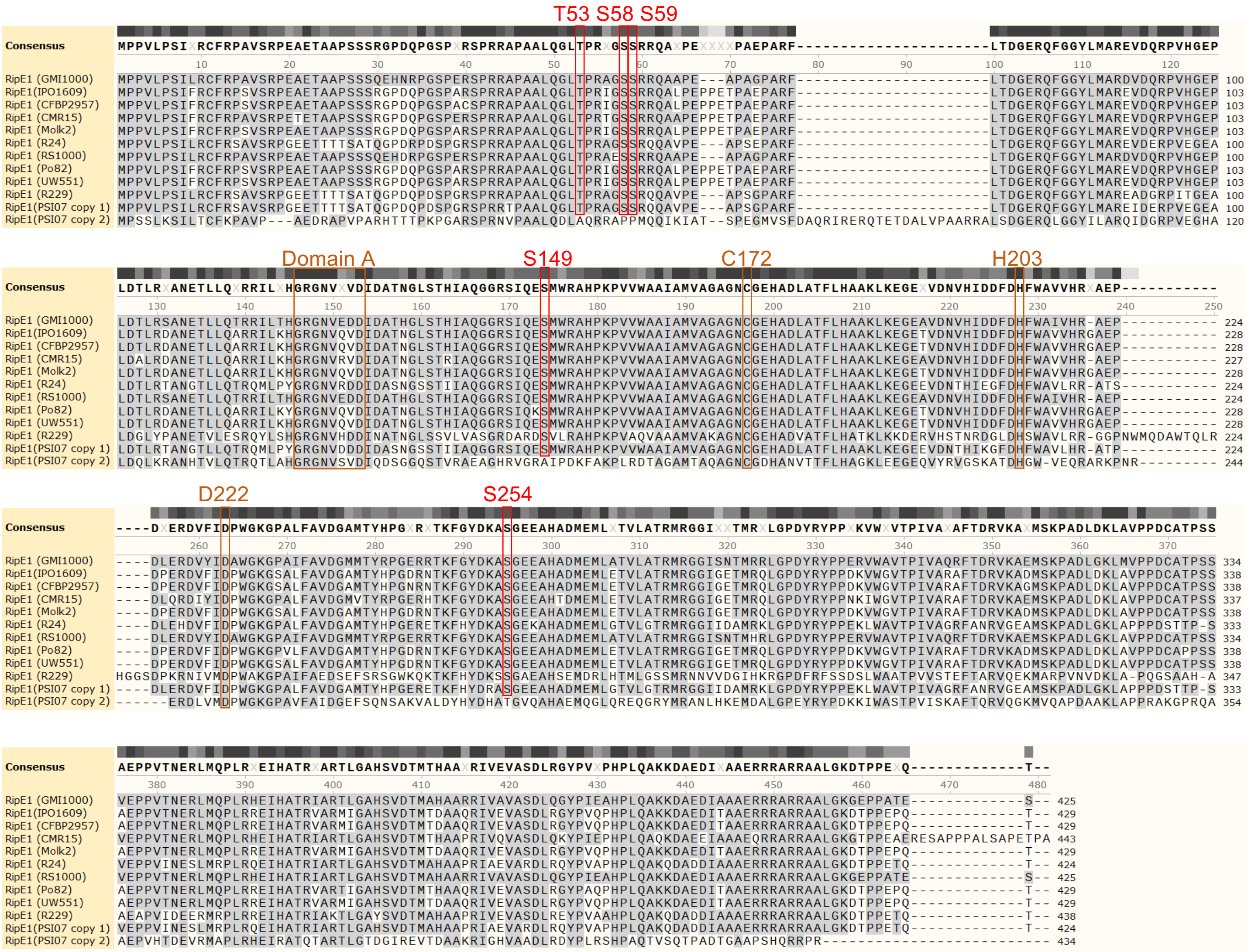

Figure S7. The phosphorylated residues in RipE1 are conserved among strains belonging to different phylotypes within the *R. solanacearum* species complex.

Protein sequence alignment showing RipE1 versions in different *R. solanacearum* strains. The phosphorylated residues analyzed in this study are indicated in red, together with the conserved domain A, and the catalytic sites.
