# Supplementary material for: A bacterial type III effector hijacks plant ubiquitin proteases to evade degradation
Source: PLoS Pathog. 2025 Jan 22;21(1):e1012882. doi: 10.1371/journal.ppat.1012882 (PMC11771917; doi:10.1371/journal.ppat.1012882)
Supplement: S2 Table — (DOCX) [file ppat.1012882.s009.docx]

| **Table S2 Primers used in this study** | | | |
| --- | --- | --- | --- |
| **Gene** | **Forward primers** | **Reverse primers** | |
| **Primers for site-directed mutagenesis** | | |  |
| *RipE1 T53A* | TGCAAGGCCTGGCGCCACGGGCC | GGCCCGTGGCGCCAGGCCTTGCA | |
| *RipE1*  *S58AS59A* | GCCACGGGCCGGAGCGGCGCGCCGGCAAGCG | CGCTTGCCGGCGCGCCGCTCCGGCCCGTGGC | |
| *RipE1 S149A* | CACGCCACATGGCTTCCTGGATGGAGCG | CGCTCCATCCAGGAAGCCATGTGGCGTG | |
| *RipE1 S254A* | CCTCGCCGGCAGCCTTGTCGTAGCCGA | TCGGCTACGACAAGGCTGCCGGCGAGG | |
| *RipE1 T53DS58DS59D* | acccacgggccggagacgacCGCCGGCAAGCGGCACCC | gtctccggcccgtgggtcCAGGCCTTGCAGGGCAGC | |
| *RipE1*  *S149D* | TCCAGGAAgacATGTGGCGTGCCCATCCC | CCACATgtcTTCCTGGATGGAGCGGCC | |
| *RipE1 S254D* | TACGACAAGGCTgacGGCGAGGAAGCCCACGCCGA | CCgtcAGCCTTGTCGTAGCCGAACTT | |
| **Primers for qRT-PCR in *N. benthamiana*** | | | |
| *RipE1* | CGGAGAACGAAGTTCGGCTA | GGCGCATGGTATTGCTGATG | |
| *RipAA* | GCCAGTACTTCAGCGTGACA | GTGCTGGTCGGGATAAACAT | |
| *eGFP* | CACATGAAGCAGCACGACCTT | AGTTCACCTTGATGCCGTTC | |
| *NbEF1a* | CCCAAGAGGCCCTCAGACA | CACACGACCAACAGGGACAGT | |
| *NbPR-1* | GGTCAACACGGCGAAAACC | GCCTTAGCAGCCGTCATGA | |
| *NbUTH05* | ACAAGACCGGATGCTGAACT | TCGACTATGAGGACCCAGGT | |
| *NbUTH12* | CAATACTGGACCTTGCGTGC | CAGTGTCTCGCCCTCATGAA | |
| *NbUTH15* | GTACTGTGGGTGACGTGCTT | TATCTCCTCTGCCCGCAATG | |
| *NbSGT1* | TAAAACCTCGGGAAATGTCG | TCTTGGCTGGTATTCCCTTG | |
| *NbPtr1* | AAAACAGGTGGACGGACTTG | TTGTCCTCCCCTACAAATGC | |
| **Primers to generate the RNAi constructs for *NbUTH* gene silencing** | | | |
| *UTH05* | CACCTGATGTGTATGCTTCTTTTGACAAG | TACCTGTGCAGAGTATAGAGATTGC | |
| *UTH12* | CACCTTGCAGCTTGATGTCAAAGG | AACACTTCGATCTGCATCAGG | |
| *UTH15* | CACCGCAACACATATGATGAAGTAGTGGA | TAGGCAATCTTATGTTCAGAATTTC | |

| **Continued Table S2 Primers used in study** | | |
| --- | --- | --- |
| **Primers for cloning** | | |
| *UTH12-FLAG* | GGAGAGGACAGGGTACCCGGGATGACTCTCATGACTCCTCCTCCA | CGAACTAGTGTCGACTCTAGAGTTGTAGATCCGAACGGGCTT |
| *UTH15-FLAG* | CATTTCATTTGGAGAGGACAGATGACCATACTGAATTCTCAACC | AGTGTCGACTCTAGAGGATCCGTTGTATATCTTGACAGGCTTTTC |
| *UTH15-GFP* | CTTGCTCACCATGGTACTAGTGTTGTATATCTTGACAGGCTTTTCAAA | CTTGCTCACCATGGTACTAGTGTTGTATATCTTGACAGGCTTTTCAAA |
| *pER8:RipE1* | ctagtcgactctagcctcgagATGCCGCCCGTCCTGCCG | gggaggcctggatcgactagtTTACTTGTACAGCTCGTCCATGCC |
