# Supplementary material for: A bacterial type III effector hijacks plant ubiquitin proteases to evade degradation
Source: PLoS Pathog. 2025 Jan 22;21(1):e1012882. doi: 10.1371/journal.ppat.1012882 (PMC11771917; doi:10.1371/journal.ppat.1012882)
Supplement: Attachment — Submitted filename: RipE1 response.docx [file ppat.1012882.s012.docx]

Dear Editor,

Thank you and the reviewers for your positive feedback. We have addressed the final comments, including moving the phosphor-mimic data in Figure 1, and we keep the figure 8 as a graphic summary. Please find below a detailed response to each comment. We have submitted a revised version of the manuscript with tracked changes, and we look forward to your final feedback to the revised version.

On behalf of all authors,

Alberto Macho

----

**Reviewers' Comments:**

Reviewer's Responses to Questions

**Part I - Summary**
Please use this section to discuss strengths/weaknesses of study, novelty/significance, general execution and scholarship.

Reviewer #1: The authors have thoroughly addressed the reviewers' comments, significantly improving the manuscript and meeting the conditions for acceptance. Regarding Figure 8, while I remain unconvinced of its scientific value as previously noted in my initial review, I defer to the editor's judgment on whether to retain or remove the figure from the main article.

Response: following the editor´s advice on this matter, we have kept the Figure 8 as a graphical summary of this work.

Reviewer #2: The authors have carefully and thoroughly revised the manuscript in response to the previous peer review comments and have provided clear responses to the concerns raised. The revised manuscript is worthy of acceptance.

**Part II – Major Issues: Key Experiments Required for Acceptance**
Please use this section to detail the key new experiments or modifications of existing experiments that should be absolutely required to validate study conclusions.

Generally, there should be no more than 3 such required experiments or major modifications for a "Major Revision" recommendation. If more than 3 experiments are necessary to validate the study conclusions, then you are encouraged to recommend "Reject".

Reviewer #1: (No Response)

Reviewer #2: None in particular

**Part III – Minor Issues: Editorial and Data Presentation Modifications**
Please use this section for editorial suggestions as well as relatively minor modifications of existing data that would enhance clarity.

Reviewer #1: (No Response)

Reviewer #2: The data for the newly added phosphorylated mutant RipE1 (RipE1 5D) is considered to be important for this manuscript, so it should be included in Figure 1 rather than in the supporting figures.

Response: following the reviewer´s advice, we now show the data for RipE1 5D in Figure 1.

PLOS authors have the option to publish the peer review history of their article ([what does this mean?](https://track.editorialmanager.com/CL0/https:%2F%2Fjournals.plos.org%2Fplospathogens%2Fs%2Feditorial-and-peer-review-process%23loc-peer-review-history/1/010f019413e443fc-b4f3091b-5c80-4982-ae29-0d809a160c95-000000/ZF8CMQ5_Wd-Hf7KXyUGuVFj2VN4Wjj8b3CZ0eziCYLo=191)). If published, this will include your full peer review and any attached files.

If you choose “no”, your identity will remain anonymous but your review may still be made public.

**Do you want your identity to be public for this peer review?** For information about this choice, including consent withdrawal, please see our [Privacy Policy](https://track.editorialmanager.com/CL0/https:%2F%2Fwww.plos.org%2Fprivacy-policy/1/010f019413e443fc-b4f3091b-5c80-4982-ae29-0d809a160c95-000000/8Vgsc-eHmXZKxc86MIgniKVgo22olDhD-tncU68xYoA=191).

Reviewer #1: No

Reviewer #2: No

Response: we thank the reviewers for their positive feedback and useful suggestions to our manuscript.
