# Supplementary material for: A bacterial type III effector hijacks plant ubiquitin proteases to evade degradation
Source: PLoS Pathog. 2025 Jan 22;21(1):e1012882. doi: 10.1371/journal.ppat.1012882 (PMC11771917; doi:10.1371/journal.ppat.1012882)
Supplement: Attachment — Submitted filename: Response to reviewers.docx [file ppat.1012882.s011.docx]

**Additional Editor Comments (if provided):**

Dear Alberto Macho,
Many thanks for submitting your manuscript on RipE1 stability. This works highlights a new aspect of co-evolution between plants and pathogens and is therefore interesting and relevant for the community.
I agree with the reviewers that this is a well written robust manuscript and consider that addressing their minor suggestions would indeed help polishing the manuscript. Finally, seconding reviewer II, I also suggest to include data on the degradation of phosphomimic versions of RipE1 as this would add robustness to the results.
Best regards,
Mariana Schuster

Response: Thank you and the reviewers for your positive feedback and useful suggestions, which have certainly contributed to improve our manuscript. We have addressed the minor suggestions and also included the data on the degradation of phosphomimic versions of RipE1, as requested. Please find our responses to the Reviewers´ comments below. We have submitted a revised version of the manuscript with tracked changes, and we look forward to your final feedback to the revised version.

On behalf of all authors,

Alberto Macho

----

**Reviewers' Comments:**

Reviewer's Responses to Questions

**Part II – Major Issues: Key Experiments Required for Acceptance**
Please use this section to detail the key new experiments or modifications of existing experiments that should be absolutely required to validate study conclusions.

Generally, there should be no more than 3 such required experiments or major modifications for a "Major Revision" recommendation. If more than 3 experiments are necessary to validate the study conclusions, then you are encouraged to recommend "Reject".

Reviewer #1: None identified.

Reviewer #2: RipE1 5A is considered to be structurally unstable and thus more susceptible to ubiquitination in the Discussion, but how would a phosphorylation mimetic mutation in RipE1 (e.g., RipE1 5D or 5E) affect RipE1 degradation? This experiment is not strictly necessary, but depending on the results, it may help to improve the conclusions of this manuscript.

Response: We thank the reviewer for this suggestion. We have generated a RipE1-5D mutant to mimic phosphorylation in these residues, and analyzed its stability compared to RipE1 WT and the RipE1-5A mutant. Interestingly, although wild-type RipE1 is stabilized by phosphorylation in plant cells, the RipE1-5D phosphomimic version showed a slightly stronger accumulation (new Figures S1C-E), further supporting the notion that a negative charge in these residues (constitutive in this case) contributes to RipE1 stability.

**Part III – Minor Issues: Editorial and Data Presentation Modifications**
Please use this section for editorial suggestions as well as relatively minor modifications of existing data that would enhance clarity.

Reviewer #1: My only comment, which is quite minor, concerns the speculation developed in the discussion about the reason for this stability of RipE1, which is only considered to benefit the pathogen. Could we conceive that RipE1's action on its virulence targets is only transient, and that an increase in RipE1 stability through phosphorylation or de-ubiquitination would favor its detection by Ptr1 to lead to HR? In terms of plant defense, this scenario would be more advantageous (as it would result in a stronger response) than a 'simple' degradation of ubiquitinated RipE1 by the proteasome. Related to this, I find Figure 8 not very enlightening for the reader, merely recapitulating the data without providing a truly explanatory or speculative vision.

Response: We thank the reviewer for suggesting this alternative scenario. We have included this possibility in the revised discussion section. However, we should keep in mind that such mechanism would only be reasonable in the context of RipE1-triggered immunity, which, to date, has only been described in *N. benthamiana*.

Regarding Figure 8, we indeed use it to recapitulate the data and provide a hypothetical working model. If the reviewer or the editor consider appropriate, this figure can be moved to the supporting materials.

Reviewer #2: None in particular.
